# Electrostatic Discharge During Helicopter Hoist Operations: A Nationwide Prospective Study of the Norwegian Search and Rescue Service

**DOI:** 10.1016/j.acepjo.2026.100468

**Published:** 2026-07-18

**Authors:** Jostein Rødseth Brede, Arnfinn Storli, Stein Bastian von Tangen-Jordan, Lasse Coucheron

**Affiliations:** 1 Department of Anaesthesiology and Intensive Care Medicine, St. Olav’s University Hospital, Trondheim, Norway; 2 Department of Emergency Medicine and Pre-Hospital Services, St. Olav’s University Hospital, Trondheim, Norway; 3 Norwegian Air Ambulance Foundation, Department of Research and Development, Oslo, Norway; 4 330 Squadron, Royal Norwegian Air Force, Sola Air Base, Norway

**Keywords:** HEMS, search and rescue, SAR, static, electrostatic discharge, winch operation, hoist operation

## Abstract

**Objectives:**

In helicopter search and rescue (SAR), rescue hoists or static underslung lines are frequently used for patient access. Electrostatic charges accumulated by the aircraft can discharge through hoisted health care personnel, a phenomenon known as electrostatic discharge (ESD) or "static." These discharges can potentially cause serious injury, yet there is a surprising scarcity of published data on their incidence and clinical impact. We aimed to determine the incidence and severity of ESD during hoist operations within the Norwegian SAR service.

**Methods:**

We conducted a nationwide, 1-year prospective observational study including all SAR bases operated by the 330 Squadron in Norway, between February 1, 2025, and January 31, 2026. All SAR hoist missions and training exercises conducted over the 1-year period were included. Data collected included ESD occurrence, hoist mission characteristics, atmospheric conditions, and subjective/objective severity of the discharge.

**Results:**

Of the 839 hoist operations performed during the study period, ESD occurred in 28 of these, representing an overall incidence of 3.3%. The risk of ESD was significantly higher during hoists over water compared with land (odds ratio [OR] 2.18, 95% CI 1.01-4.70). There was precipitation within 5 nautical miles in 71% of ESD incidents, and grounding equipment was found inadequate in 64%. Severity was classified as moderate in 29% (n = 8) and severe in 21% (n = 6) of ESD incidents. Two rescue paramedics required hospital admission. ESD caused communication equipment failure in 11% (n = 3) of incidents and led to the termination of the hoist operation in 18% (n = 5) of the incidents.

**Conclusion:**

This nationwide study demonstrates that ESD occurs in 3.3% of hoist operations performed by the SAR service in Norway. With half of the ESD incidents rated as moderate or severe and a notable frequency of mission terminations and hospitalizations, ESD represents a significant occupational hazard. These findings highlight an urgent need to re-evaluate safety protocols for SAR personnel, and to further investigate the scope and health implications of ESD for SAR personnel.

**The Bottom line**

In this nationwide, 1-year prospective study, electrostatic discharge occurred in 3.3% of helicopter hoist operations, with the risk doubling over water. Half of these incidents were classified as moderate or severe, leading to significant operational and clinical consequences, such as equipment failures, mission terminations, and hospital admissions for search and rescue personnel. These findings highlight an urgent need to re-evaluate safety protocols to protect flight crews during hoist operations.

## 1 Introduction

### 1.1 Background

Helicopter emergency medical services (HEMS) are a critical component of modern health care systems, providing essential care for severely ill or injured patients across Europe and the United States.^1^, ^2^, ^3^, ^4^ Use of HEMS is particularly important when it reduces time for health care workers to reach a patient or shortens transport time to definitive care.^4^^,^^5^ Furthermore, aerial transport is indicated when ground access is restricted by challenging terrain, such as offshore, mountainous, or remote locations. In Norway, the civilian HEMS use a static, underslung rope procedure to access patients, whereas the search and rescue (SAR) service use a rescue hoist.^6^^,^^7^ These operations are similar to many other services in Europe and both civilian and military services in the United States.^8^^,^^9^

**Figure 1:**
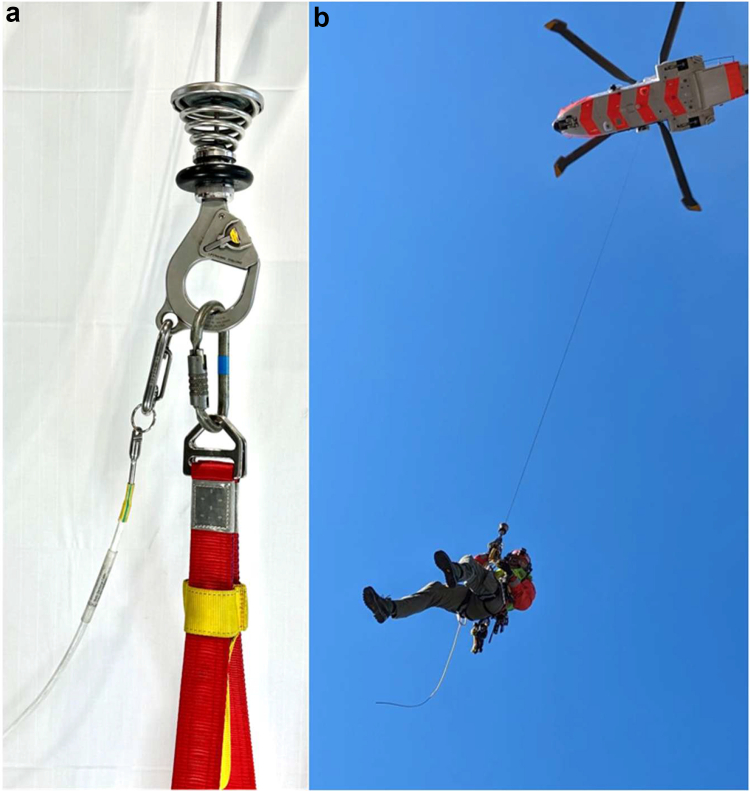
Hoist hook and standard grounding equipment. Figure 1a: a discharge line (left) and a rescue strop attached to the hoist hook. Figure 1b: the discharge line is visible hanging below the rescue paramedic during a hoist operation.

It is well established that helicopters accumulate significant electrostatic charges during flight.^10^, ^11^, ^12^, ^13^, ^14^, ^15^ Electrostatic discharge (ESD) can cause direct injury through muscle contractions, cardiac arrhythmias, cellular damage, or trauma to the nervous system. It also poses indirect risks, in which syncope or a fall can lead to secondary accidents (Video 1).^16^^,^^17^ To mitigate these risks to hoisted personnel, a discharge line is used to ground the aircraft (Fig.1). Ideally, this line should make initial contact with the surface during hoist operations. Despite these protocols, rescue paramedics (RP) and other involved flight crew (eg, physicians) in Norway frequently report ESD incidents, often referred to as "static."^18^ It is likely that ESD is both well-known and relevant for other HEMS and SAR services globally.

### 1.2 Importance

Medical literature lacks systematic descriptions of the incidence and severity of ESD. Anecdotal reports from HEMS and SAR services suggest significant injuries and hospitalizations among personnel. A recent nationwide survey in Norway indicated that severe ESD is a common experience among SAR personnel, with concerns for potential long-term health consequences.^18^

### 1.3 Goals of This Investigation

We aimed to prospectively investigate the incidence and classify the severity of ESD incidents in the Norwegian SAR service through a nationwide, 1-year study, and to evaluate factors associated with ESD incidents.

## 2 Methods

### 2.1 Study Setting

Seven SAR helicopter bases operate throughout mainland Norway, in addition to a SAR base on Svalbard and SAR helicopters stationed on selected offshore oil rigs. These bases conduct more than 2500 missions annually.^19^ Six of the 7 mainland SAR bases are governed by the Justice Department and operated by 330 Squadron, Royal Norwegian Air Force.^7^ The 330 squadron operates a Leonardo AW101 helicopter with a 6-person crew: 2 pilots, a system operator, a flight engineer/hoist operator, an RP, and a consultant anesthesiologist. All hoist operations standardly involve either an RP and/or a physician. The RP is the primary rescue resource, and the physician is frequently involved in hoisting, similar to other HEMS systems.^6^^,^^7^^,^^20^^,^^21^ Physicians are included in all hoist operations, except rough sea pick-up or when patients are extracted directly from water. In cases where the hoist operation is presumed to be particularly challenging, the RP is often the only hoisted resource.

### 2.2 Study Design

This was a nationwide prospective observational study. Data were registered between February 1^st^, 2025, and January 31^st^, 2026, via the web-application *Nettskjema* (www.nettskjema.no), designed and operated by the University Information Technology Center at the University of Oslo. The application meets the privacy requirements in Norway.^22^ The registration form was designed by the first author (JRB) and tested by the other authors for fail-proof and logical design.

The study was performed at the 6 Norwegian SAR helicopter bases in Norway operated by the 330 squadron, as well as the SAR training base at Sola, Norway, also operated by the 330 squadron. These bases perform most of the hoist operations over land and coastal regions in Norway. Inclusion of all bases in the 330 squadron ensured a nationwide study.^19^ All hoist operations, by either a RP or a physician, and including both actual missions and for training purposes, were registered. After a hoist operation was completed, the RP or physician scanned a QR-code that led to the registration form. Some questions were only applicable if ESD incidents were experienced. All ESD incident registrations and measurements of severity and mission consequence were based on self-reporting. All the RP and physicians had previously participated in a survey on ESD experience, and the ESD severity grading was based on the classification used in that study.^18^ All RPs and physicians were therefore familiar with the measurement of severity; additionally a definition catalog of this severity was available on all bases during the study period. There were 33 RPs and 48 physicians working at the 330 Squadron during the study period and data was anonymously registered. An email with a reminder of study inclusion and study progression was sent to all RPs 3 times during the study period.

The study is reported in accordance with the Strengthening the Reporting of Observational Studies in Epidemiology statement guidance.^23^

### 2.3 Statistical Analysis

Categoric variables are described as count and/or proportion (%). For categoric variables Fisher exact test is used. A *P*< .05 (2-tailed) is regarded as statistically significant. Associations between environmental and operational factors were quantified using the Phi (ϕ) coefficient. To account for the nonlinear relationship of temperature to ESD risk, temperature was analyzed as a categoric variable with 3 distinct intervals: > +3 degrees, between −3 and +3 degrees, and < −3 degrees. These intervals were treated as independent binary variables (dummy coded) to permit calculation of specific correlation coefficients against all other binary factors. To visualize the complex interactions across these dimensions, 2 mapping techniques were applied to the resulting correlation matrix. First, a network analysis used a force-directed spring layout to identify clusters of variables that move together. Second, multidimensional scaling (MDS) was employed to project the variable associations into a 2D spatial map, where the distance between nodes represents the dissimilarity (d = 1 - ϕ) of the factors. Closer proximity in the MDS map indicates a higher frequency of co-occurrence. All analyses were performed using Python (v3.10) with the *scikit-learn*, *networkx*, and *seaborn* libraries. Statistical analysis was performed with SPSS (IBM Corp. Released 2017. IBM SPSS Statistics for Windows, Version 25.0. IBM Corp).

### 2.4 Ethics Approval and Consent to Participate

The study was approved by the Regional Committee for Medical and Health Research Ethics (reference 861938 REK-Midt) and by the flight insurance officer at the 330 squadron, Royal Norwegian Air Force.

## 3 Results

**Figure 2:**
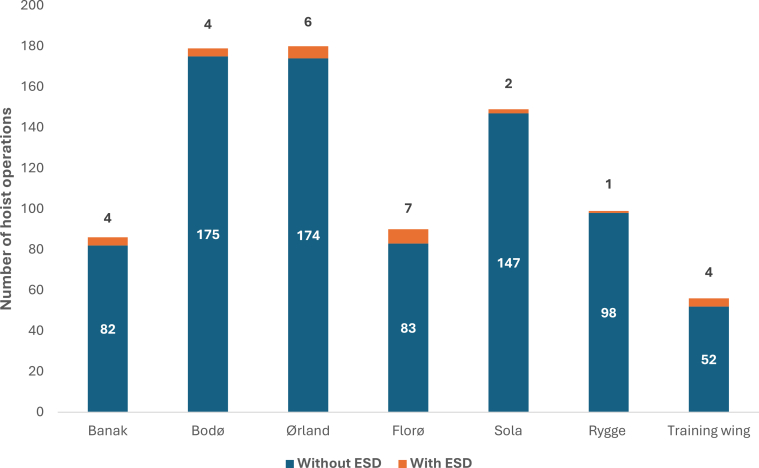
Distribution of total hoist operations and ESD incidents across bases. ESD, electrostatic discharge.

During the 1-year study period, 839 hoist operations were performed, of which 324 (38.6%) were conducted over water and 515 (61.4%) over land. ESD was registered in 28 of the 839 operations, resulting in an overall incidence of 3.3%. The distribution of total hoist operations and ESD incidents across the different bases is shown in Figure 2.

The environmental and operational characteristics of the 28 recorded ESD incidents are detailed in Table 1.

**Table 1:** Characteristics of ESD incidents.

Operational and environmental factors	Total incidents = 28
	n (%)
Environment	
Hoist over water	16 (57.1)
Hoist over land	12 (42.9)
Precipitation closer than 5 nautical miles	
Yes	20 (71.4)
No	8 (28.6)
Temperature	
>3 °C	19 (67.9)
−3 °C to +3 °C	3 (10.7)
<3 °C	6 (21.4)
Discharge line contacted ground before ESD	
Yes	18 (64.3)
No	10 (35.7)
ESD considerations were performed prior to hoisting	
Yes	12 (42.9)
No	16 (57.1)
Guideline used	
Yes	16 (57.1)
No	12 (42.9)

Percentages are calculated based on the total number of incidents. Although 57.1% of the 28 incidents happened over water, the absolute incidence rate over water was 4.9% (16/324) compared with 2.3% over land (12/515).

ESD, electrostatic discharge.

**Figure 3:**
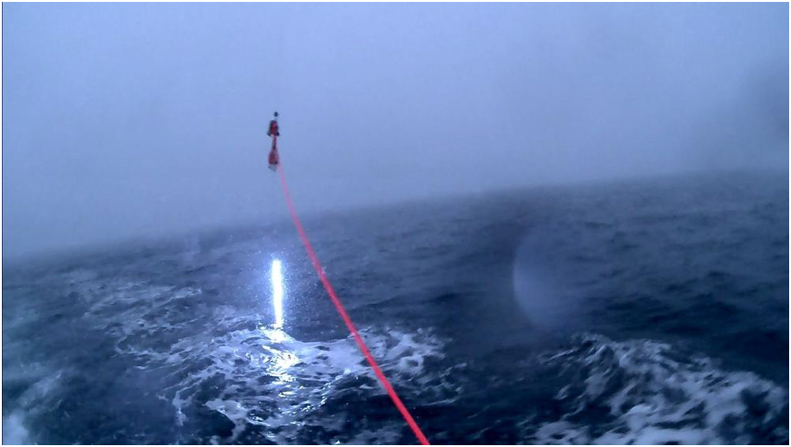
An approximately 80 cm long electrostatic discharge arc seen during a naval hoist operation.^18^

Subjective experiences and clinical consequences during the ESD incidents are described in Table 2. A visible electrical arc was observed in 8 incidents (28.6%) (Fig 3, Video 2), and a loud audible bang was reported in one incident (3.6%). Notably, in 5 of the incidents (17.9%), the ESD event resulted in operative consequences, with immediate termination of hoist operation.

**Table 2:** Subjective experiences and clinical consequences of ESD incidents.

Experiences during ESD incident	Total incidents = 28
	n (%)
Visual/auditory phenomena	
Visible electrical arc	8 (28.6)
Loud audible bang	1 (3.6)
Clinical severity	
Mild grade of ESD	19 (67.9)
Tingling or light shock in arms/fingers	14 (50)
Shock in legs during landing	5 (17.9)
Moderate grade of ESD	8 (28.6)
Communication equipment failure	3 (10.8)
Severe shock in arms or body	4 (14.2)
Shock to face	1 (3.6)
Severe grade of ESD	6 (21.4)
Powerful shock to head/face causing a fall	2 (7.1)
Temporary paralysis	1 (3.6)
Syncope or unconsciousness	1 (3.6)
Examined by physician or admitted to hospital	2 (7.1)
Subjective pain/discomfort rating	
Slightly uncomfortable or negligible	14 (50)
Very uncomfortable	10 (35.7)
Severely painful	4 (14.2)
Operational consequence	
No	23 (82.1)
Yes, hoist operation terminated	5 (17.9)

One ESD incident may include more than one grade of clinical severity. Percentages are calculated from the total number of ESD incidents.

ESD, electrostatic discharge.

**Figure 4:**
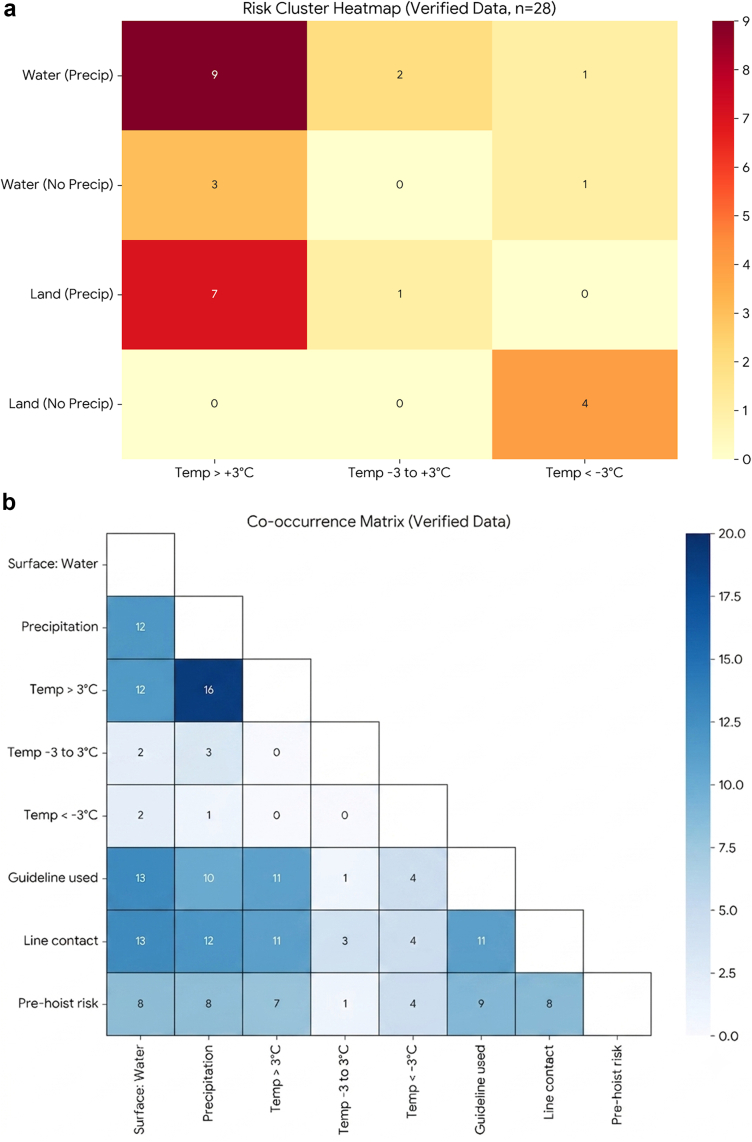
Risk cluster heatmap for the 28 electrostatic incidents. Figure 4a: Heatmap based on environmental contexts. The vertical axis categorizes incidents by surface type and the presence of precipitation. The horizontal axis segments the incidents in 3 temperature categories. Cell values represent the absolute frequency, with the color gradient indicating increasing incident density. Figure 4b: Co-occurrence matrix based on both operational and environmental risk factors. Each cell represents the total count of incidents where both factors were present.

**Supplemental figure 1:**
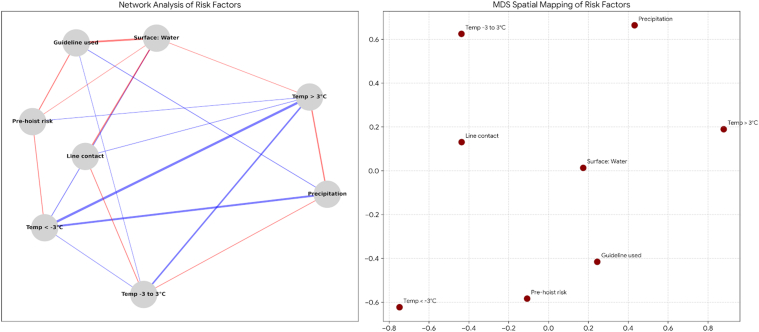
Multidimensional mapping of risk profiles. (Left) Network Analysis: A graph-based representation where nodes represent risk factors and edges represent the strength of interaction (based on correlation). Red lines indicate positive associations, while blue lines indicate negative associations; edge thickness is proportional to the interaction strength. (Right) Multi-Dimensional Scaling: A spatial mapping where the distance between points reflects the similarity in how risk factors behave across the data set. Factors plotted in proximity suggest a high degree of dependency, whereas isolated factors represent independent risk contributors. MDS indicates multidimensional scaling.

The incidence of ESD was significantly higher during hoists over water (4.9%) compared with over land (2.3%) (OR 2.18; 95% CI 1.01-4.70). A multivariate interaction matrix demonstrating how environmental and operational factors converged during these incidents is shown in Figure 4. A multidimensional mapping of risk profiles is shown in Figure S1. There was no missing data in the registered data set.

## 4 Limitations

Our study has limitations. The SAR service in Norway operates predominantly in coastal and mountainous areas, where precipitation (ie, snow, hail, and rain) is frequent. These findings may therefore not be fully transferable to inland services with more stable weather conditions. Additionally, data collection was based on voluntary self-reporting. It is likely that uneventful hoist operations (without ESD) were underreported, hence our calculated overall incidence of 3.3% may represent an overestimation. However, it is likely that the actual ESD incidents are reliably registered as they are easily remembered. We did not record environmental factors for hoist operations without ESD, hence a comparison if these missions characteristics are unattainable.

## 5 Discussion

This nationwide prospective study demonstrates that ESD is experienced in 3.3% of all hoist operations within the Norwegian SAR service. The ESD was classified as moderate or severe in approximately half of the incidents and led to termination of the ongoing hoist operation in 18% of incidents.

Although most hoist operations were performed over land, the incidence of ESD was significantly higher during hoists over water. The multivariate analysis (Fig 4) indicates that the risk of ESD is likely a product of several converging environmental factors rather than an isolated variable. The most critical risk cluster identified is hoisting over water in temperatures exceeding +3 °C during conditions with active precipitation. This “maritime precipitation cluster” accounted for 9 of the 28 reported ESD incidents (32.1%). This may be attributable to the electromagnetic environment on the sea surface, where downwash from the rotor over saltwater creates a dense suspension of aerosolized brine. This “salt spray” may increase the conductivity of the air column surrounding the hoist cable, narrowing the dielectric gap between the aircraft and the sea, and create a low-resistance path to ground upon contact.^15^

Notably, the static discharge line contacted ground prior to the ESD incident in 18 of 28 times (64.3 %), indicating that standard grounding protocols are frequently insufficient. This failure may be caused by triboelectric charging rates.^10^, ^11^, ^12^, ^13^, ^14^ that exceed the dissipation capacity of the equipment, inherent limitations in the standard grounding system, or insulating surface conditions (such as snow or ice) that prevent the grounding mechanism of the discharge line. Additionally, the ambient electric field strength may be significantly enhanced if the helicopter operates near convective clouds, such as Cumulonimbus formations.^24^^,^^25^

As a single risk variable, precipitation was present in 20 out of 28 incidents (71.4%). This correlates well with increased triboelectric charging, as rotor blades hit particles in the air. Prehoist ESD risk assessments were performed in 12 of the 28 incidents (42.8%). However, we did not register this data for uneventful hoist operations in which static was not experienced; hence, it is unknown how common such prehoist assessments are. Based on our findings, we argue that an operational risk assessment for ESD should prioritize precipitation status.

Nineteen of the registrations on subjective experiences were considered mild severity, 8 were of moderate severity, and 6 were severe (Table 2, Video 1), with some incidents involving multiple overlapping symptoms. Notably, 2 RPs required physician examination or hospital admission within the 1-year study period. Considering the limited number of personnel exposed, this represents a concerning frequency of clinically significant consequences. Furthermore, the ESD-induced failure of communication equipment is a critical operational hazard, as it drastically increases the risk of secondary, unwanted incidents during complex rescues.

Our results demonstrate that ESD is an occupational hazard in the Norwegian SAR service, consistent with findings from a recent cross-sectional survey assessing prior ESD experience among SAR personnel.^18^ In that survey, 100% of the RP had experienced ESD, including personnel with only 2 years of service experience. Even though this was a study from Norway, we believe that the findings are generalizable to other countries with HEMS or SAR services that use hoist or fixed rope, particularly those that operate in coastal areas, areas with much precipitation or in dry, sandy environments. These are relevant conditions for many other European countries, as well as both the civilian and military HEMS and SAR services in the United States.^8^^,^^9^

This nationwide prospective study demonstrates that ESD, or “static”, is an occupational hazard for personnel during helicopter hoist operations, with an incidence of 3.3%. The highest risk for ESD occurs during hoists over water, in temperatures exceeding +3 °C, and in the presence of precipitation. With half of the incidents classified as moderate or severe, and standard grounding equipment failing to prevent discharge in most incidents, these findings highlight an urgent need to re-evaluate the technical ESD mitigation strategies and the long-term health implications for SAR personnel.

## Author Contributions

JRB designed and planned the study. AS, SBTJ and LC contributed to the conduct of the study. JRB drafted the article and prepared the tables and figures. All authors revised the manuscript and have read and approved the final version of the manuscript.

## Funding and Support

By *JACEP Open* policy, all authors are required to disclose any and all commercial, financial, and other relationships in any way related to the subject of this article as per ICMJE conflict of interest guidelines (see www.icmje.org). This work was supported by the 10.13039/501100013232Norwegian Air Ambulance Foundation. The funder had no role in the conceptualization, design, data collection, analysis, or preparation of the manuscript.

## Conflict of Interest

All authors have affirmed they have no conflicts of interest to declare.

## Supplementary Materials

Video 1 Video of a hoist operation where the rescue paramedic is hit by the discharge line and receives an electrostatic discharge that causes a syncope and fall.

Video 2 A naval hoist operation, where an approximately 80 cm long electrostatic discharge arc is seen.
